# Highly directional single-photon source

**DOI:** 10.1515/nanoph-2023-0276

**Published:** 2023-07-12

**Authors:** Alejandro Manjavacas, F. Javier García de Abajo

**Affiliations:** Instituto de Óptica (IO-CSIC), Consejo Superior de Investigaciones Científicas, 28006 Madrid, Spain; Department of Physics and Astronomy, University of New Mexico, Albuquerque, NM 87106, USA; ICFO-Institut de Ciencies Fotoniques, The Barcelona Institute of Science and Technology, 08860 Castelldefels, Barcelona, Spain; ICREA-Institució Catalana de Recerca i Estudis Avançats, Passeig Lluís Companys 23, 08010 Barcelona, Spain

**Keywords:** lattice resonances, periodic arrays, quantum emitters, single-photon source

## Abstract

Single-photon emitters are a pivotal element in quantum technologies, but the generation of single photons along well-defined directions generally involves sophisticated configurations. Here, we propose a photon source capable of generating single photons with high efficiency along guided modes. Specifically, we consider a quantum emitter placed in a periodically patterned linear waveguide. The latter is designed to host a single guided mode over the spectral range of interest and display a divergence in the photonic density of states at an emission wavelength close to the period. Photons are preferentially emitted along the waveguide near that spectral region. We predict that nearly all of the emission can be made to occur along the waveguide with a reduction in the temporal uncertainty by two orders of magnitude. Our study opens a conceptually new direction in the production of single photons with a high degree of directionality and reduced temporal uncertainty.

## 1 Introduction

Single photons (SPs) are a fundamental ingredient of quantum-optics devices with applications in quantum computing, cryptography, and metrology [1–3]. Deterministic sources are capable of producing SPs at designated times by first exciting a molecule or a quantum dot (QD) with close to 100 % probability (e.g., in a Λ excitation scheme), which later decays into a photon within a characteristic time of the order of a few to tens of nanoseconds [1, 4, 5]. In contrast to the classical Poissonian statistics obtained from a dimmed conventional source, the quantum nature of SPs is well confirmed by anti-bunching experiments [6] and Hong–Ou–Mandel interferometry [7]. The quality of a SP source relies on its ability (1) to extract photons with large efficiency, (2) to reduce the uncertainty in the emission time, (3) to deliver photons at a high repetition rate, and (4) to rule out two-photon events. These criteria depend strongly on the types of emitter and dielectric environment, which currently allow ruling out two-photon events in practice, with repetition rates beyond MHz, and temporal uncertainties of the order of ns [8, 9]. The extraction efficiency and the uncertainty in emission time can be improved via the Purcell effect [10] by placing the emitter in a nanostructured environment, which, in turn, helps to increase the indistinguishability of the emitted photons [8, 11, 12]. However, capturing all photons along a one-dimensional (1D) channel remains a challenge that has been partially addressed by complicated setups [13–16], although emission in a high-index planar waveguide already gives good confinement in 2D [9, 17, 18].

In this article, we present a new class of photon source producing highly directional emission, which can in principle be brought arbitrarily close to 100 % collection efficiency. We consider an emitter placed inside a waveguide with a single guided mode to which the emission is preferentially coupled [19]. By periodically structuring the waveguide, this mode acquires zero group velocity near the boundary of the first Brillouin zone, which is accompanied by a divergence in the photonic local density of states (LDOS). This divergence places a dominant weight on the emission through the guided mode in realistic designs. Similar results have been obtained in waveguides patterned in photonic crystal slabs [20, 21], but with the requirement of an extended 2D structure to sustain the guided mode [9, 22, 23]. We theoretically demonstrate that our scheme is robust against random imperfections of the waveguide and reduces the temporal uncertainty in the emission by two orders of magnitude relative to the free emitter.

**Figure 1:**
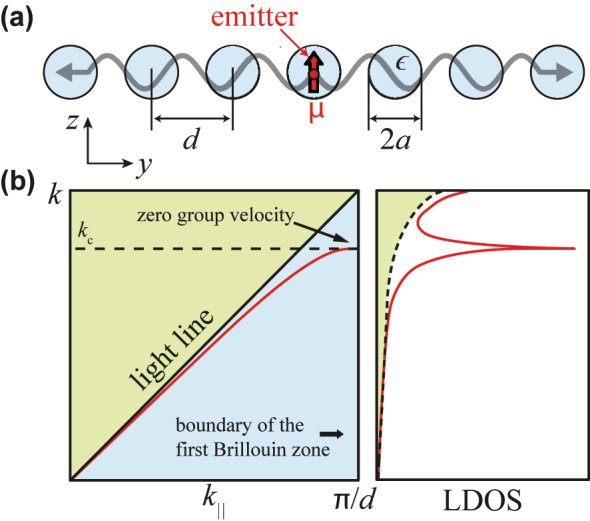
Description of the underlying mechanism of our directional photon source. (a) A photon emitter is placed inside a particle within a linear array of identical particles. (b) The array displays a guided mode that shows up to the right of the line cone (i.e., with parallel wave vector *k*_‖_ larger than the free-light wave vector *k*) and has zero group velocity at the boundary of the first Brillouin zone (*k*_‖_ = *π*/*d*) in parallel wave-vector space (left). The LDOS receives contributions from spontaneous emission into the light cone and from coupling to the guided mode of the array (right). The latter exhibits a van Hove singularity associated with the vanishing group velocity, and thus, it dominates the overall emission.

We consider a periodic array of dielectric particles, as shown in Figure 1(a), with an emitter located at the center of one of the particles. The photonic structure of the array is schematically depicted in Figure 1(b). Provided the permittivity of the spheres *ϵ* is larger than the environment (vacuum in our case), a guided mode of frequency *ω* exists to the right of the light cone in the dispersion diagram as a function of light wavenumber *k* = *ω*/*c* and the wave vector component parallel to the direction of the array *k*_‖_ (see below). As it is usually found in periodic systems, the group velocity of this mode vanishes at the boundary of the first Brillouin zone (*k*_‖_ = *π*/*d*, where *d* is the period of the array). At this point, it is useful to consider the LDOS, defined as the sum of the position-dependent near-field intensity associated with all modes for a given frequency [24]. The emission rate is known to be proportional to the LDOS [25]. In our structure, the LDOS exhibits a van Hove singularity [26] at the frequency for which the mode has zero group velocity [Figure 1(b)], and this directly translates into a divergent emission probability. We must emphasize that the increased rate near this point originates in the contribution of the guided mode, and therefore, the emission is predominantly confined to the array.

## 2 Theoretical description

For simplicity, we assume that the particles in the linear array are small compared to the period [radius *a* ≪ *d*, see Figure 1(a)], so that they are described through their polarizability *α* (full inclusion of multipoles leads to similar results, see Supplementary Materials, Figure S1). The latter is obtained from the electric-dipole Mie coefficient [27] to correctly account for retardation at the level of single-particle scattering (see Methods). We take the emitter dipole *μ* to be oriented perpendicularly to the array, and thus, all induced dipoles and fields are also perpendicular and the problem becomes one-dimensional because this is a polarization-preserving scheme. The dipole induced at each particle *j* is then given by the self-consistent coupled equations

(1) $$p_{j}=p^{\text{em}}\delta_{j0}+\alpha \underset{j^{\prime }\neq j}{\sum }G_{j-j^{\prime }}p_{j^{\prime }}.$$

Here, *G*_*j*−*j*′_ = e^*ikr*^(*k* ^2^/*r* + *ik*/*r* ^2^ − 1/*r* ^3^) is the dipole–dipole interaction between particles *j* and *j*′ [28], separated by a distance *r* = |*j* − *j*′|*d*. The emitting particle *j* = 0 produces a feeding dipole *p* ^em^, related to the emitter dipole *μ* as *p* ^em^ = *T* ^in^ *μ*, where the transmission coefficient *T* ^in^ is readily calculated from Mie theory (see Methods). For finite arrays, we solve Eq. (1) by direct matrix inversion.

We also present results for infinite arrays, which are dealt with by going to Fourier-transform space as ${\tilde{p}}_{k_{\|}}=\sum_{j}p_{j}{\text{e}}^{-ik_{\|}dj}$ . This allows us to write the solution

(2) $${\tilde{p}}_{k_{\|}}={\left(1-\alpha {\tilde{G}}_{k_{\|}}\right)}^{-1}p^{\text{em}}.$$

A guided mode is predicted by Eq. (2) subject to the condition $\alpha^{-1}={\tilde{G}}_{k_{\|}}$ . For lossless particles and *k*_‖_ > *k* (i.e., outside the light cone), both sides of this equation have identical imaginary parts given by −2*k* ^3^/3 [29]. The remaining real parts are also equal along the dispersion curve of Figure 1(b) [i.e., along the guided mode signaled by a zero of the denominator in Eq. (2)].

For an emitter of transition dipole *μ*, the emission rate inside a homogeneous dielectric of permittivity *ϵ* can be obtained from the far-field Poynting vector divided by the photon energy [30], leading to $\sqrt{\epsilon }\;\Gamma_{0}$ , where Γ_0_ = 4*k* ^3^|*μ*|^2^/3*ℏ* is the emission rate in free space. This expression must be corrected by the fact that the emitter is not permeated by the *ϵ* material. This correction allows us to treat real emitters such as small molecules, rare earth ions, or vacancy centers, for which the transition dipole is constructed out of the initial and final electronic (or vibrational) states, but the surrounding homogeneous medium does not permeate inside them. Assuming a point-like emitter placed in an arbitrarily small spherical empty cavity, the corresponding local-field correction factor becomes [31] *f* = 3*ϵ*/(2*ϵ* + 1). In addition, the presence of material boundaries produces a reflected field *E* ^ref^ acting back on the emitter and modifying the emission rate. The latter then becomes (see Ref. [30] for a detailed derivation)

(3) $$\Gamma =|f{|}^{2}\left(\Gamma_{0}\sqrt{\epsilon }+2\hbar^{-1}Im\left\{\mu^{*}E^{\text{ref}}\right\}\right),$$

where we introduce the aforementioned local-field correction. In our system, *E* ^ref^ is produced by the emitting sphere surface and the rest of the spheres in the array. By symmetry, this field is aligned with the emission dipole. When the emitting particle is isolated from the rest of the array, we have *E* ^ref^ = *R* ^in^ *μ*, which produces Γ = Γ_iso_ when inserted into Eq. (3). For the array, we have $E^{\text{ref}}=R^{\text{in}}\mu +T^{\text{out}}\left(p_{0}-p^{\text{em}}\right)$ , where the reflection and transmission coefficients *R* ^in^ and *T* ^out^ are also derived from Mie theory (see Methods), and *p*_0_ − *p* ^em^ is the dipole induced in the emitting sphere by scattering from the rest of the particles. Combining the above expressions, the decay rate in the infinite array admits the expression

(4) $$\begin{array}{ll} \Gamma & =\Gamma_{\text{iso}}+\frac{2|f{|}^{2}|\mu {|}^{2}d}{\pi \hbar }\\ & \;\times Im\left\{T^{\text{out}}T^{\text{in}}\overset{\pi /d}{\underset{0}{\int }}\frac{\text{d}k_{\|}}{{\left(\alpha {\tilde{G}}_{k_{\|}}\right)}^{-1}-1}\right\}. \end{array}$$

The integral in Eq. (4) contains a pole due to the guided mode. It is an integrable divergence, except near the mode cutoff, *k* = *k*_c_ [see Figure 1(c)]. The emission rate can thus be made arbitrarily large by choosing a photon frequency close to that point. Importantly, we have assumed that the dipole moment of the emitter is perpendicular to the array (i.e., in the *xz* plane). However, any small unintended component of the dipole along the direction of the array can degrade the coupling by emitting into free-space radiation (e.g., we expect a fractional reduction of the coupling given by the square of the misalignment angle for small values of the latter). Therefore, to achieve a high degree of performance in the proposed approach, it is crucial to obtain a good alignment of the dipole perpendicular to the array.

## 3 Results and discussion

### 3.1 Fraction of guided emission

**Figure 2:**
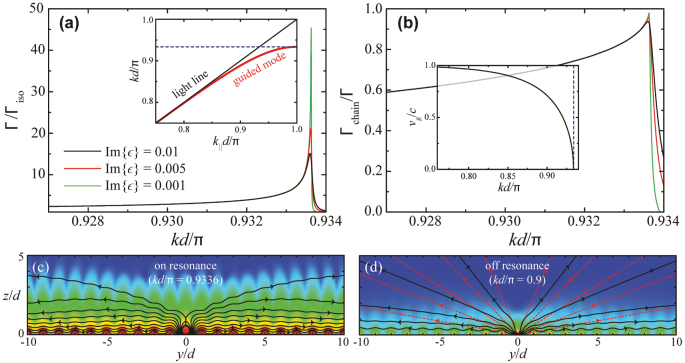
Directional emission of the single-photon source. (a) Dependence of the emission rate Γ from an excited emitter placed at the center of one of the particles in the linear array of Figure 1(a) as a function of emission wave vector *k*. The rate is normalized to the value Γ_iso_ obtained when the particle is isolated (i.e., far from the rest of the array). The inset shows the dispersion relation of the guided mode in the array. (b) Fraction of emission along the guided mode. The group velocity is shown in the inset. (c) and (d) On- and off-resonance near electric-field intensity on a common color scale. Poynting-vector streamlines are superimposed on the contour plots (solid curves) and compared to those for an isolated sphere [dashed curves in (d)]. The particles have radius *a* = 0.3 *d*, where *d* is the period of the array, and they are assumed to have a permittivity of real part Re{*ϵ*} = 14 and small imaginary part [see labels in (a)]. The emission dipole is normal to the array direction.

We consider silicon spheres in the infrared region (*ϵ* ≈ 14). The ratio of sphere radius to array period is set to *a*/*d* = 0.3. Figure 2(a) shows the total emission rate Γ obtained from Eq. (4) and normalized to Γ_iso_. A small imaginary part has been added to the permittivity, as indicated by labels. As expected, the emission increases dramatically near the cutoff wave vector *k*_c_ ≈ 0.934*π*/*d*, reaching over an order of magnitude faster rates compared to the emitter placed in an isolated particle for realistic values of Im{*ϵ*}. The maximum rate increases with decreasing absorption, and it diverges in the Im{*ϵ*} = 0 limit. This is beneficial for the source because it reduces the uncertainty in the time of emission.

The fraction of emission that is guided along the waveguide (Γ_chain_) is represented in Figure 2(b), as obtained from the contribution of the pole of Eq. (4) for *k*_‖_ > *k*, divided by the total emission rate. It is also peaked at *k*_*c*_, where it reaches 97 % for Im{*ϵ*} = 0.001. In the absence of absorption, 100 % of the emission ends up in the guided mode. Simultaneously, the group velocity *v*_g_ goes to zero at *k*_c_ [Figure 2(b), inset], although it rapidly rises at lower values of *k* while the fraction of guided emission is still above 90 %.

The near-field patterns of Figure 2(c) and (d) reveal a dramatic difference between on- and off-resonance emissions, with the former clearly showing a large field intensity being guided along the array, whereas the Poynting vector streamlines indicate a larger relative contribution of decay into freely propagating light in the off-resonance scenario.

### 3.2 Temporal uncertainty in the emission

**Figure 3:**
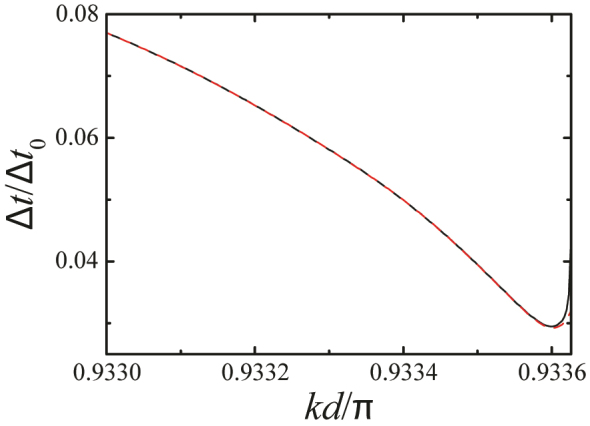
Uncertainty in the emission time Δ*t* under the conditions of Figure 2 for *d* = 0.5 μm, normalized to the uncertainty for the emitter placed in vacuum, Δ*t*_0_. The solid curve includes the uncertainty introduced by the propagation of the emitted photon over a distance of 20 mm along the array, considering the change in group velocity over the frequency width of the emission.

A potential problem with our scheme of SP generation lies in the temporal uncertainty introduced by the group velocity dispersion within the spectral width of emission, since the source is operating near a region of zero group velocity. This effect is shown to contribute negligibly in Figure 3, where the total temporal uncertainty Δ*t* is represented with (solid curve) and without (dashed curve) inclusion of the different delay produced by the group velocity along a 20 mm-long waveguide (see Methods for more details). In fact, Δ*t* is considerably reduced relative to the uncertainty for the emitter in vacuum (Δ*t*_0_) as a result of the increase in Γ [i.e., Δ*t*/Δ*t*_0_ ≈ Γ_0_/Γ, see also Figure 2(a)].

### 3.3 Effect of disorder and the finite size of the array

**Figure 4:**
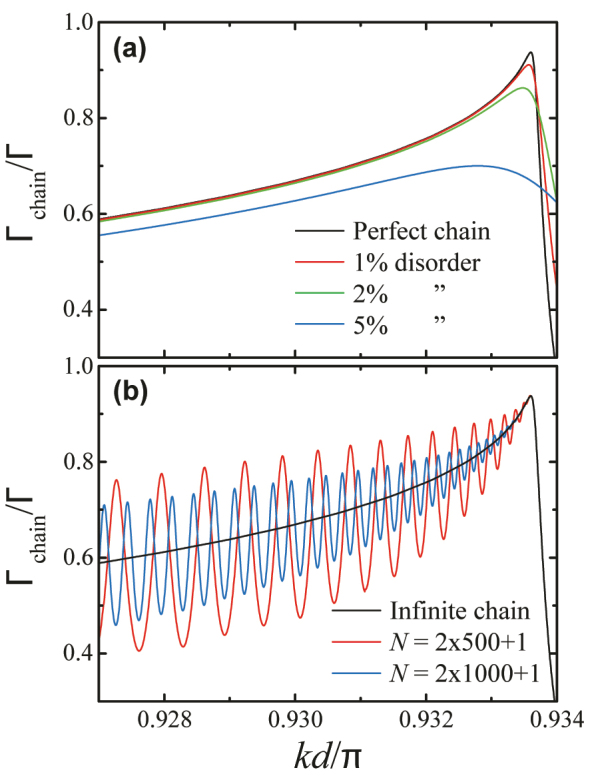
Effect of disorder and the finite size of the array. (a) Fraction of emission along the guided mode of the array under the same conditions as in Figure 2, but including the effect of disorder, which is introduced through a phenomenological model explained in the main text for various degrees of random variations in the particle radii and positions (expressed as a percentage of the radius and period, respectively). (b) Finite-size effects for arrays consisting of 1001 and 2001 particles, compared to an infinite array.

Fabrication imperfections can lead to a dramatic loss of coherence in the scattering from different particles within the array. This effect can be effectively described by adding an extra term to Im{*ϵ*} ∼ (*ϵ* − 1)(*ϵ* + 2)[(*δa*)^2^/*a* ^2^ + *k* ^2^(*δd*)^2^/18], as proposed elsewhere [32] from a Debye–Waller analysis of the effect of an average random variation in particle radius *δa* and inter-particle distance *δd*. In this way, the outcoupling of the guided mode from the waveguide is effectively described via an equivalent absorption in the material of the particles. Following this approach, we find that over 90 % of the emission is still guided for a disorder (*δa*)/*a* = (*δd*)/*d* = 1 %, and even values above 80 % are found for 2 % disorder, and 70 % for 5 % disorder [see Figure 4(a)]. We have also studied the effect of misplacement of the emitter away from the center of the spherical particle and found <10 % reduction in coupling efficiency for displacements by *λ*/100 (see Supplementary Materials, Figure S2).

Finite-size effects in the array can also damage the performance of the source, particularly through Fabry–Perot resonances of the guided mode due to reflection at the boundaries of the particle chain. To estimate this effect in a phenomenological way, we employ the discrete Fourier transform for finite chains, thus resulting in discretized values of *k*_‖_, the sum over which must be substituted for the integral in Eq. (4). The contribution of *k*_‖_ > *k* (Γ_chain_) is then associated with guided modes, which are eventually either absorbed or outcoupled from the chain by scattering at the ends. Notice that absorption in the chain and, consequently, the propagation length are both roughly proportional to the inverse of the imaginary part of the dielectric function of the particles. We present the results of this analysis in Figure 4(b) for arrays consisting of 1001 and 2001 spheres, as compared to the infinite array. The guided fraction near *k*_c_ is well preserved, but Fabry–Perot resonances are observed at lower frequencies, with a period in *k* inversely proportional to the number of particles. This period decreases near *k*_c_ as a result of adiabatic compression for vanishing group velocity, which effectively cancels out the effect of the noted resonances right at *k*_c_: the ends of the array can only be reached in infinite time for *v*_g_ = 0.

## 4 Conclusions

We have proposed a scheme for a highly directional photon source based upon periodically corrugated waveguides, capable of collecting nearly all of the emission from a quantum emitter placed inside it. Actually, there is no fundamental limit for reaching full guided emission, which can be obtained by optimization of geometrical parameters. By symmetry, photons are emitted in our proposed scheme with equal probability along both directions of the waveguide. However, different strategies can be adopted to emit photons in only one direction. For instance, circularly polarized emitters could be used by exploiting optical spin–orbit coupling [33]. Another possibility consists in altering the position of several particles at one of the ends of the chain to build a mirror in the same way as a distributed Bragg reflector. Furthermore, our waveguide design is robust against random imperfections in the size and position of its periodic elements. Additionally, it produces a significant reduction in the temporal uncertainty of the emission. The choice of materials is not critical, as silica particles produce similar results to those reported here for silicon spheres, and large bandwidths can also be achieved (see Supplementary Materials, Figure S3).

When compared to other approaches, we note that the proposed 1D structure has a smaller footprint than 2D photonic crystals, thus presenting an advantage in terms of integration. The guided mode is automatically confined to a 1D channel, in contrast to 2D configurations. We thus conceive the design of 1D waveguides in which emitters can be activated at different locations, and with waveguide interconnects relying on local rearrangements of the scattering particles to maximize coupling to the desired waveguide route. A three-dimensional (3D) structure incorporating 1D waveguides would thus be a possible architecture for optimum light routing, to which the present study contributes with a design for a single-photon generator having good quantum efficiency.

Although, for simplicity and tutorial purposes, we have considered that the waveguides are formed by spherical scatterers, our analysis can be straightforwardly applied to more general kinds of scatterers (e.g., other scatterer morphologies). In particular, an appealing possibility consists in creating defects or periodic corrugations in a waveguide (for example, through ion implantation or via lithography alongside a waveguide of cylindrical or rectangular cross section). Another possibility would be to use two-photon photolithography to pattern scatterers in 3D systems (e.g., the 3D interconnected 1D waveguides mentioned above), although the inclusion of emitters can be challenging in this configuration. Nanoparticle self-assembly is yet another possibility, which, for example, can rely on specific Mie modes of high-index dielectric particles arranged in a chain, so that guided modes are obtained from the hybridization of such modes. In general, high-order modes of arbitrary order and polarization nature could be employed to construct guided modes. In this approach, emitters could be inserted in individual spheres, acting as emission centers. Actually, each particle could be decorated with an emitter in a configuration such that light with higher photon energy is used to excite only one of them (e.g., by non-resonantly addressing a single scatterer, exploiting the smaller diffraction-limited focal spot that can be produced at a higher photon energy).

As an interesting possibility, the noted divergence in the LDOS, which is responsible for the enhanced guided emission, could also be used to strongly couple distant quantum emitters sitting at different sites along the waveguide, suggesting a possible application in quantum devices.

## 5 Methods

### 5.1 Transmission and reflection coefficients for a dielectric sphere

The electric field in a homogeneous medium of permittivity *ϵ* can be expressed in terms of electric and magnetic scalar functions *ψ* ^E^ and *ψ* ^M^ as [34, 35] ***E*** = ***L*** *ψ* ^M^ − (*i*/*k*)∇ × ***L*** *ψ* ^E^, where ***L*** = −*i* ***r*** × ∇ is the angular momentum operator relative to the origin, *k* is the free-space light wavenumber, and we consider a unity magnetic permeability. In our system, we only need to consider the electric component, whose associated scalar function is found to satisfy the wave equation $\left(\nabla^{2}+k_{\epsilon }^{2}\right)\psi^{\text{E}}=0$ , where $k_{\epsilon }=k\sqrt{\epsilon }$ . This implies that the scalar function can be expanded in multipolar components (orbital and azimuthal numbers *l* and *m*) given by $j_{l}\left(k_{\epsilon }r\right)Y_{l,m}\left(\hat{r}\right)$ and $h_{l}^{\left(+\right)}\left(k_{\epsilon }r\right)Y_{l,m}\left(\hat{r}\right)$ in terms of spherical harmonics *Y*_*l*,*m*_, and spherical Bessel and Hankel functions *j*_*l*_ and $h_{l}^{\left(+\right)}$ . Here, we consider fields oriented along *z* and dominated by a dipolar term, so that only the *l* = 1 and *m* = 0 component is involved. More precisely, in regions free from external sources, we have

(A5a) $$\psi^{\text{E}}\left(r\right)=\;j_{1}\left(k_{\epsilon }r\right)Y_{1,0}\left(\hat{r}\right)\psi_{1,0}^{\text{E}},$$

whereas the field produced by a dipolar source at the origin corresponds to a scalar function

(A5b) $$\psi^{\text{E}}\left(r\right)=\;h_{1}^{\left(+\right)}\left(k_{\epsilon }r\right)Y_{1,0}\left(\hat{r}\right)\psi_{1,0}^{\text{E}}.$$

For a unit dipole, the field must read $E=\left(k_{\epsilon }^{2}\hat{z}+\nabla \partial_{z}\right)e^{ik_{\epsilon }r}/\epsilon r^{3}$ . Upon direct inspection of the involved functions, we find that this expression coincides with Eq. (A5b) for a coefficient $\psi_{10}^{\text{E,dip}}=\;k^{3}\sqrt{4\pi /3}$ . Likewise, an electric field *E* along *z* is produced at the origin when a coefficient

(A6) $$\psi_{10}^{\text{E,uni}}=\sqrt{3\pi /\epsilon }\;E$$

is inserted into the scalar function of Eq. (A5a).

When a dipole is placed at the center of a free-standing homogeneous sphere of permittivity *ϵ* and radius *a*, the scalar function becomes

(A7a) $$\begin{array}{ll} \psi^{\text{E}} & =\left(h_{1}^{\left(+\right)}\left(k_{\epsilon }r\right)Y_{1,0}\right.+\left.r_{1}^{\text{E}}j_{1}\left(k_{\epsilon }r\right)Y_{1,0}\right)\psi_{10}^{\text{E,dip}},\;\;\left(r<a\right) \end{array}$$

(A7b) $$\psi^{\text{E}}=t_{1}^{\text{E,in}}h_{1}^{\left(+\right)}\left(kr\right)Y_{1,0}\psi_{10}^{\text{E,dip}},\;\left(r>a\right)$$

where the first term in Eq. (A7a) accounts for the dipolar source field, the second term corresponds to the field produced upon reflection at the sphere surface, and Eq. (A7b) describes the field outside the sphere. The reflection and transmission coefficients $r_{1}^{\text{E}}$ and $t_{1}^{\text{E,in}}$ are determined by imposing the electromagnetic boundary conditions at the sphere surface, namely, the continuity of both *ϵψ* ^E^ and $\left(1+r\partial_{r}\right)\psi^{\text{E}}$ [34], which lead to

(A8a) $$r_{1}^{\text{E}}=\frac{h_{1}^{\left(+\right)}\left(\rho \right){\left[\rho_{\epsilon }h_{1}^{\left(+\right)}\left(\rho_{\epsilon }\right)\right]}^{\prime }-\epsilon h_{1}^{\left(+\right)}\left(\rho_{\epsilon }\right){\left[\rho h_{1}^{\left(+\right)}\left(\rho \right)\right]}^{\prime }}{\epsilon j_{1}\left(\rho_{\epsilon }\right){\left[\rho h_{1}^{\left(+\right)}\left(\rho \right)\right]}^{\prime }-h_{1}^{\left(+\right)}\left(\rho \right){\left[\rho_{\epsilon }j_{1}\left(\rho_{\epsilon }\right)\right]}^{\prime }},$$

(A8b) $$\;t_{1}^{\text{E,in}}=\epsilon \frac{j_{1}\left(\rho_{\epsilon }\right){\left[\rho_{\epsilon }h_{1}^{\left(+\right)}\left(\rho_{\epsilon }\right)\right]}^{\prime }-h_{1}^{\left(+\right)}\left(\rho_{\epsilon }\right){\left[\rho_{\epsilon }j_{1}\left(\rho_{\epsilon }\right)\right]}^{\prime }}{\epsilon j_{1}\left(\rho_{\epsilon }\right){\left[\rho h_{1}^{\left(+\right)}\left(\rho \right)\right]}^{\prime }-h_{1}^{\left(+\right)}\left(\rho \right){\left[\rho_{\epsilon }j_{1}\left(\rho_{\epsilon }\right)\right]}^{\prime }},$$

where *ρ* = *ka*, *ρ*_*ϵ*_ = *k*_*ϵ*_ *a*, and the prime indicates differentiation with respect to these variables.

Likewise, an external dipolar field component scattered by the sphere is represented by the scalar function

(A9a) $$\psi^{\text{E}}=\left(j_{1}\left(\rho \right)Y_{1,0}+t_{1}^{\text{E,scat}}h_{1}^{\left(+\right)}\left(\rho \right)Y_{1,0}\right)\psi_{10}^{\text{E}},hom,\;\;\;\;\left(r>a\right)$$

(A9b) $$\psi^{\text{E}}=t_{1}^{\text{E,out}}j_{1}\left(\rho_{\epsilon }\right)Y_{1,0}\psi_{10}^{\text{E,hom}},\;\;\;\left(r<a\right)$$

where the first term in Eq. (A9a) represents the incident field, the second term accounts for the scattered field, and Eq. (A9b) describes the field transmitted inside the sphere. Applying again the boundary conditions, we find

(A10a) $$t_{1}^{\text{E,scat}}=\frac{-j_{1}\left(\rho \right){\left[\rho_{\epsilon }j_{1}\left(\rho_{\epsilon }\right)\right]}^{\prime }+\epsilon j_{1}\left(\rho_{\epsilon }\right){\left[\rho j_{1}\left(\rho \right)\right]}^{\prime }}{h_{1}^{\left(+\right)}\left(\rho \right){\left[\rho_{\epsilon }j_{1}\left(\rho_{\epsilon }\right)\right]}^{\prime }-\epsilon j_{1}\left(\rho_{\epsilon }\right){\left[\rho h_{1}^{\left(+\right)}\left(\rho \right)\right]}^{\prime }},\;$$

(A10b) $$\;t_{1}^{\text{E, out}}=\frac{h_{1}^{\left(+\right)}\left(\rho \right){\left[\rho j_{1}\left(\rho \right)\right]}^{\prime }-j_{1}\left(\rho \right){\left[\rho h_{1}^{\left(+\right)}\left(\rho \right)\right]}^{\prime }}{h_{1}^{\left(+\right)}\left(\rho \right){\left[\rho_{\epsilon }j_{1}\left(\rho_{\epsilon }\right)\right]}^{\prime }-\epsilon j_{1}\left(\rho_{\epsilon }\right){\left[\rho h_{1}^{\left(+\right)}\left(\rho \right)\right]}^{\prime }}$$

for the coefficients in Eqs. (A9a) and (A9b).

From the above analysis, a dipole *μ* oriented along *z* and placed at the center of a self-standing sphere of radius *a* and permittivity *ϵ* has an associated scalar function given by Eqs. (A9a) and (A9b) with $\psi_{10}^{\text{E,dip}}=k^{3}\sqrt{4\pi /3}\;\mu$ . The scalar function component associated with the transmitted field (outside the sphere) can be obtained by multiplying this expression by the coefficient $t_{1}^{\text{E,in}}$ [Eq. (A8b)], and therefore, when observed from the exterior of the sphere, the dipole appears to have an effective magnitude *p* ^em^ = *T* ^in^ *μ* with $T^{\text{in}}=t_{1}^{\text{E,in}}$ . Likewise, the field reflected inside the sphere has an associated scalar function with a coefficient $\psi_{10}^{\text{E,ref}}=k^{3}\sqrt{4\pi /3}\;r_{1}^{\text{E}}\mu$ , corresponding to a uniform field *E* ^ref^ = *R* ^in^ *μ* with $R^{\text{in}}=\left(2k^{3}/3\right)\sqrt{\epsilon }\;r_{1}^{\text{E}}$ [see Eqs. (A6) and (A8a)].

The presence of other spheres surrounding the one that contains the quantum emitter produces a self-induced field acting like an additional external field given by $E_{\text{ext}}^{\text{ind}}=\alpha^{-1}\left(p_{0}-p^{\text{em}}\right)$ , which is associated with an *incident* scalar function $\psi_{10}^{\text{E,ind}}=\sqrt{3\pi }\alpha^{-1}\left(p_{0}-p^{\text{em}}\right)$ according to Eq. (A6). Multiplying this expression by $t_{1}^{\text{E, out}}$ [see Eqs. (A9b) and (A10b)], we obtain the corresponding scalar function inside the sphere. Finally, using Eq. (A6) again, the field associated with the latter is simply given by $E^{\text{ind}}=T^{\text{out}}\left(p_{0}-p^{\text{em}}\right)$ , where $T^{\text{out}}=t_{1}^{\text{E, out}}\sqrt{\epsilon }\alpha^{-1}$ with $t_{1}^{\text{E, out}}$ given by Eq. (A10b).

The particle polarizability is calculated from the Mie coefficient in Eq. (A10a) as $\alpha ={t_{1}}^{\text{E,scat}}/(2k^{3}/3)$ .

### 5.2 Temporal uncertainty in the emission

The temporal uncertainty in the emission of our system (Δ*t*) is the result of the interplay between a reduction produced by the enhancement in the emission rate (Δ*t*_Γ_) and an increase associated with larger group velocities $\left(\Delta t_{v_{\text{g}}}\right)$ . We can write

$$\frac{\Delta t}{\Delta t_{0}}=\frac{\Delta t_{\Gamma }+\Delta t_{v_{\text{g}}}}{\Delta t_{0}},$$

where Δ*t*_0_ is the temporal uncertainty for the emitter in vacuum. The first term in this expression is inversely proportional to the emission rate (i.e., Δ*t*_Γ_/Δ*t*_0_ = Γ_0_/Γ). The second term accounts for the fact that different spectral components travel with different velocities, thus widening the photon pulse and increasing the temporal uncertainty according to

(C11) $$\Delta t_{v_{\text{g}}}=\frac{L}{v_{\text{g}}\left(\omega_{1}\right)}-\frac{L}{v_{\text{g}}\left(\omega_{2}\right)}\approx L\frac{\partial \left(1/v_{\text{g}}\right)}{\partial \omega }\Delta \omega ,$$

where Δ*ω* = *ω*_1_ − *ω*_2_ is the spectral width of the photon pulse and *L* is the distance traveled along the waveguide. Figure 3 shows that $\Delta t_{v_{g}}$ is negligible compared with Δ*t*_Γ_.

## Supplementary Material

Supplementary Material Details
