# Supplementary material for: Highly directional single-photon source
Source: Nanophotonics. 2023 Jul 12;12(16):3351–8. doi: 10.1515/nanoph-2023-0276 (PMC11501204; doi:10.1515/nanoph-2023-0276)
Supplement: Supplementary file 1 — Supplementary Material Details [file j_nanoph-2023-0276_suppl_001.pdf]

Highly directional single-photon source

– Supplementary Materials –

A. Manjavacas[1,2] and F. Javier García de Abajo[3,4]

[1] *Instituto de Óptica (IO-CSIC), Consejo Superior de Investigaciones Científicas, 28006 Madrid, Spain*
[2] *Department of Physics and Astronomy, University of New Mexico, Albuquerque, New Mexico 87106, United States*
[3] *ICFO-Institut de Ciencies Fotoniques, The Barcelona Institute of Science and Technology, 08860 Castelldefels (Barcelona), Spain*
[4] *ICREA-Institució Catalana de Recerca i Estudis Avançats, Passeig Lluís Companys 23, 08010 Barcelona, Spain*

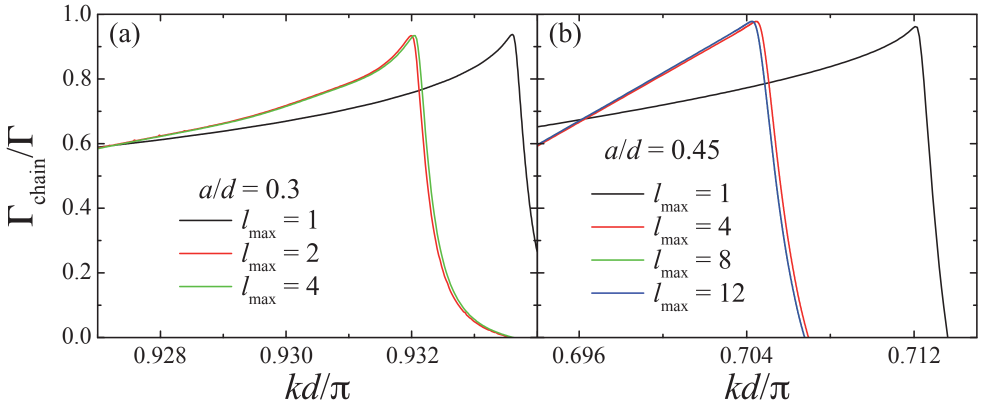

FIG. S1: **Multipolar corrections.** The theoretical model presented in the main text is limited to the dipolar approximation, which allows us to derive concise analytical formulas from which we can extract valuable direct insight. The precision of such approximation is questionable when the size of the particles becomes comparable to the wavelength. This figure compares results for the fraction of guided emission obtained by using either the dipolar approximation ($l_{\rm max} = 1$) or a rigorous solution of Maxwell's equations including higher-order multipoles up to a maximum orbital angular momentum number $l_{\rm max}$ (see labels). The ratio of the particle radius to the period of the array is $a/d = 0.3$ and $a/d = 0.45$ in (a) and (b), respectively. The particle permittivity is $\epsilon = 14 + 0.01\,{\rm i}$. For these choices of parameters, the dipolar approximation produces reasonably accurate results, apart from a slight redshift in the resonance frequency.

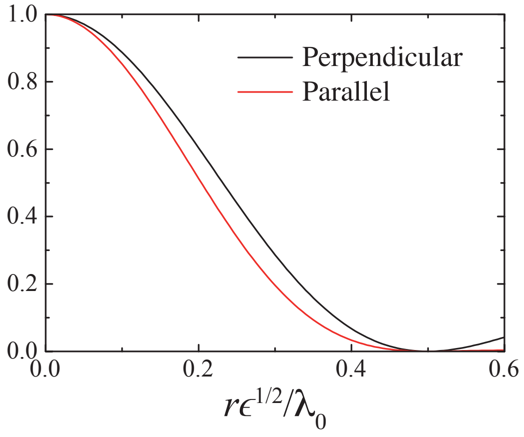

FIG. S2: **Effect of a displacement of the quantum emitter from the center of the particle.** The extraordinary performance of the proposed photon source is based upon the large induced field produced at the center of the central sphere. For this reason, a displacement of the quantum emitter from the center of the sphere can deteriorate the properties of the photon source. To study the effect of such displacement, we calculate the weight of the dipolar component in the multipolar expansion around the center of the sphere when the dipole is placed at a distance r from that point along either perpendicular (black curve) or parallel (red curve) directions relative to the array. We observe a reduction by less than 10% for distances up to $r = 0.1\,\lambda_0/\sqrt{\epsilon}$, thus emphasizing the robustness of the proposed photon source.

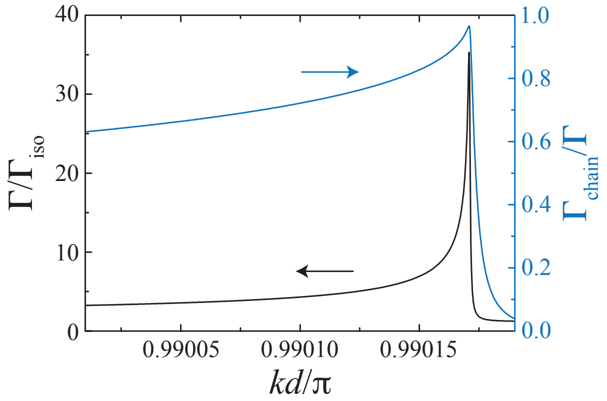

FIG. S3: **Emission rate enhancement for particles of lower permittivity.** We plot the same information as in Fig. 2(a),(b) of the main text, but for silica particles ($\epsilon = 2.1 + 9 \times 10^{-6}\,$i) with a radius-to-period ratio $a/d = 0.4$.
